# Cellular and molecular atlas of the placenta from a COVID‐19 pregnant woman infected at midgestation highlights the defective impacts on foetal health

**DOI:** 10.1111/cpr.13204

**Published:** 2022-02-09

**Authors:** Jingsi Chen, Lili Du, Feiyang Wang, Xuan Shao, Xiaoyi Wang, Wenzhe Yu, Shilei Bi, Dexiong Chen, Xingfei Pan, Shanshan Zeng, Lijun Huang, Yingyu Liang, Yulian Li, Rufang Chen, Fengwu Xue, Xiuying Li, Shouping Wang, Manli Zhuang, Mingxing Liu, Lin Lin, Hao Yan, Fang He, Lin Yu, Qingping Jiang, Zhongtang Xiong, Lizi Zhang, Bin Cao, Yan‐Ling Wang, Dunjin Chen

**Affiliations:** ^1^ 117980 Department of Obstetrics and Gynecology Key Laboratory for Major Obstetric Diseases of Guangdong Province The Third Affiliated Hospital of Guangzhou Medical University Guangzhou China; ^2^ Guangdong Engineering and Technology Research Center of Maternal‐Fetal Medicine Guangzhou PR China; ^3^ Guangdong‐Hong Kong‐Macao Greater Bay Area Higher Education Joint Laboratory of Maternal‐Fetal Medicine Guangzhou China; ^4^ State Key Laboratory of Stem Cell and Reproductive Biology Institute of Zoology Institute for Stem Cell and Regeneration Chinese Academy of Sciences Beijing China; ^5^ University of Chinese Academy of Sciences Beijing China; ^6^ 12466 Fujian Provincial Key Laboratory of Reproductive Health Research School of Medicine Xiamen University Xiamen China; ^7^ 117980 Department of General Practice The Third Affiliated Hospital of Guangzhou Medical University Guangzhou China; ^8^ 117980 Department of Infectious Diseases Key Laboratory for Major Obstetric Diseases of Guangdong Province The Third Affiliated Hospital of Guangzhou Medical University Guangzhou China; ^9^ Department of Obstetrics The First People's Hospital of Foshan Foshan Guangdong Province China; ^10^ 117980 Department of Operating Room The Third Affiliated Hospital of Guangzhou Medical University Guangzhou China; ^11^ 117980 Department of Pathology The Third Affiliated Hospital of Guangzhou Medical University Guangzhou China; ^12^ Department of Obstetrics and Gynecology Nanfang Hospital Southern Medical University Guangzhou PR China

**Keywords:** COVID‐19, immune activation, infective course, midgestation, placenta, single‐cell RNA sequencing

## Abstract

**Objectives:**

The impacts of the current COVID‐19 pandemic on maternal and foetal health are enormous and of serious concern. However, the influence of SARS‐CoV‐2 infection at early‐to‐mid gestation on maternal and foetal health remains unclear.

**Materials and methods:**

Here, we report the follow‐up study of a pregnant woman of her whole infective course of SARS‐CoV‐2, from asymptomatic infection at gestational week 20 to mild and then severe illness state, and finally cured at Week 24. Following caesarean section due to incomplete uterine rupture at Week 28, histological examinations on the placenta and foetal tissues as well as single‐cell RNA sequencing (scRNA‐seq) for the placenta were performed.

**Results:**

Compared with the gestational age‐matched control placentas, the placenta from this COVID‐19 case exhibited more syncytial knots and lowered expression of syncytiotrophoblast‐related genes. The scRNA‐seq analysis demonstrated impaired trophoblast differentiation, activation of antiviral and inflammatory CD8 T cells, as well as the tight association of increased inflammatory responses in the placenta with complement over‐activation in macrophages. In addition, levels of several inflammatory factors increased in the placenta and foetal blood.

**Conclusion:**

These findings illustrate a systematic cellular and molecular signature of placental insufficiency and immune activation at the maternal–foetal interface that may be attributed to SARS‐CoV‐2 infection at the midgestation stage, which highly suggests the extensive care for maternal and foetal outcomes in pregnant women suffering from COVID‐19.

## Graphical abstract

Here, we report the follow‐up study of a pregnant woman of her whole infective course of SARS‐CoV‐2, from asymptomatic infection at gestational week 20 to mild and then severe illness state, finally cured at Week 24 and delivering at Week 28 due to incomplete uterine rupture. The placenta from this case exhibited more syncytial knots and lowered expressions of syncytiotrophoblast‐related genes. The single‐cell RNA sequencing analysis demonstrated impaired trophoblast differentiation, activation of antiviral and inflammatory CD8 T cells, as well as the tight association of increased inflammatory responses with complement over‐activation in macrophages. These findings illustrate a systematic cellular and molecular signature of placenta insufficiency and immune activation that may be attributed to SARS‐CoV‐2 infection at midgestation stage, which highly suggests the extensive care for maternal and fetal outcomes in COVID‐19 pregnancy.

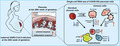

## 1 INTRODUCTION

Coronavirus disease 2019 (COVID‐19), a serious contagious disease caused by novel severe acute respiratory syndrome coronavirus 2 (SARS‐CoV‐2), has spread to over 200 countries worldwide. Although pregnant women were more susceptible to the coronaviruses and several pregnant women infected with SARS‐CoV‐2 are reported to have severe adverse outcomes,^1^, ^2^ most pregnant women underwent mild or asymptomatic infections without lasting consequences.^3^ The potential impact of SARS‐CoV‐2 infection during pregnancy on maternal and foetal development, regardless of the presence or absence of symptoms, need to be investigated with additional analysis and research.

The placenta expresses the receptors for SARS‐CoV‐2, including ACE2 and TMPRSS2, throughout pregnancy,^4^ indicating the potential spread of SARS‐CoV‐2 via the placenta to the foetus. Some investigations have confirmed transplacental transmission of SARS‐CoV‐2 at early‐to‐mid gestation and late gestation using immunohistochemistry, RNA in situ hybridization, or both, for viral antigens or viral nucleic acid in foetal cells of placenta.^5^, ^6^, ^7^, ^8^, ^9^, ^10^, ^11^, ^12^ Histological features such as maternal vascular malperfusion, foetal vascular malperfusion, chronic histiocytic intervillositis or increased intervillous fibrin were found in a small set of placentas from infected women.^13^, ^14^, ^15^, ^16^ However, transplacental transmission of the virus is very uncommon and has not been identified in most COVID‐19 pregnant cases.^17^, ^18^ It has been well known that a normal functional placenta is an effective barrier to prevent intrauterine vertical transmission of pathogens. Placental factors may be the major reason that so few placentas become infected with SARS‐CoV‐2. It is, therefore, crucial to uncover the intrinsic defence system and innate immune molecular changes of the placental barrier in COVID‐19 pregnant women.

Here, we reported a case of a COVID‐19 pregnant woman (marked as COVID‐RS in the following) infected with SARS‐CoV‐2 at the 20th week of gestation. We followed up the woman from asymptomatic, mild illness and severe illness to recovery stages until pregnancy termination due to an incomplete uterine rupture at gestational week 28. Histological analysis of the foetal and placental tissues as well as single‐cell transcriptome for the placenta was performed. No intrauterine transmission of SARS‐CoV‐2 was found in this patient, whereas the cellular and molecular signatures indicate placenta insufficiency and significant antiviral and pro‐inflammatory response at the maternal–foetal interface. The findings thus highlight the extensive care for maternal and foetal health in pregnant women suffering from COVID‐19, even after timely clinical treatment for the virus infection.

## 2 MATERIALS AND METHODS

### 2.1 Ethics approval and consent to participate

The study was approved by the Ethics Committee Medical Ethics Committee at The Third Affiliated Hospital of Guangzhou Medical University (Medical Research No. 2020090). Written informed consent for this study was obtained from the enrolled women.

### 2.2 Specimen collection

Nasopharyngeal and vaginal swabs were collected at admission and during hospitalization to test for SARS‐CoV‐2. At delivery, maternal blood, umbilical cord blood and foetal blood were collected in EDTA tubes. Full‐thickness placental biopsies were obtained. In the case of caesarean delivery, amniotic fluid was collected without blood or meconium contamination. Tissue samples from the foetus were collected after death with written informed consent from the pregnant woman. Both biopsies and blood samples were obtained sterilely by a dedicated operator. The demographic characteristics of this COVID‐RS case and the gestational age‐matched noninfection preterm patients (delivering at 29–31 weeks of gestation because of cervical dysfunction but without any other complications, labelled as CTRL group hereafter) enrolled in this study are displayed in Table S1.

### 2.3 Single‐cell isolation, library preparation and sequencing

For single‐cell RNA‐seq, cells from the placenta were isolated by using the previously described protocol.^19^ Two preterm placenta samples (details described in Table S1) were used as noninfection control. Briefly, placenta tissues were minced, enzymatically digested for 30–40 min using cocktail dissociation solution, treated with DMEM containing 10% FBS and filtered (70‐mm nylon filter; Falcon). Resuspended cell pellets were subjected to 1 × Red Blood Cell Removal Solution (Biogems) for 5 min to remove red blood cells and then subjected to be washed twice. After red blood cells lysis and dead cells removing using the Dead Cell Removal Kit (Miltenyi Biotec), cells were counted using an automatic cell counter. Then, viable cells were used for single‐cell RNAseq library construction using the Chromium Single Cell 3’ kit v2 (10× Genomics), following the manufacturer's instructions. After quantifying the DNA libraries, the single‐cell libraries’ sequencing was performed using the Illumina Platform (HiSeq X Ten System). 5′ transcriptomic sequencing data were also performed for the COVID‐RS placenta. The 3′ and 5′ transcriptomic sequencing data were both analysed as duplication. Samples were sequenced on a Novaseq 6000 with the following run parameters: read 1, 26 cycles; read 2, 98 cycles; and index 1, 8 cycles. A median sequencing depth of 25,000 reads/cell was targeted for each sample. The CellRanger (10XGenomics) analysis pipeline was used to generate a digital gene expression matrix from the sequencing data.

### 2.4 Single‐cell RNA data analysis

For placenta samples, 5′ and 3′ transcriptome sequencing data from the COVID‐RS patient and 3’ transcriptome sequencing data from 2 CTRL women with the comparable duration of gestational age were analysed. Seurat v.3 was used for downstream analysis. The following criteria were then applied to each cell of all samples: gene number between 200 and 6000 and mitochondrial gene percentage proportion < 0.05. We utilized the R package scDblFinder^20^ (version 1.9, https://github.com/plger/scDblFinder) to eliminate doublets/multiplets in single‐cell sequencing data according to the previous reports.^21^, ^22^ After filtering, a total of 17,481 cells were left for the following analysis. The datasets were integrated using the standard Seurat v3 integration workflow. The graph‐based clustering was performed using FindCluster in Seurat v.3.

### 2.5 Differential gene expression analysis and Gene functional annotation

Wilcox in Seurat v.3 (FindAllMarkers function) was used to perform differential gene expression analysis. For each cluster of placenta cells, differentially expressed genes (DEGs) of COVID‐RS placenta were generated to compare with the same cell type of the CTRL group. A gene was considered significant with adjusted Bonferroni‐adjusted *p* < 0.05. Gene Ontology (GO) enrichment analyses of differentially expressed genes were implemented by the ClusterProfiler R package. GO terms with corrected *p*‐value < 0.05 were considered significantly enriched by differentially expressed genes. Dot plots were used to visualize enriched terms by the enrichplot R package.

### 2.6 Cell–cell interaction analysis

CellPhoneDB^23^ (version2.0, https://github.com/Teichlab/cellphonedb) used the cell type annotation and counts from our single‐cell transcriptomics data to compute cell–cell communication within the identified cell subtypes between COVID‐RS case and CTRL group. The default ligand–receptor pair information was used in this process. The *p* value < 0.05 indicated significant enrichment of the interacting ligand–receptor pair in each of the interacting pairs of cell subpopulations and Cellchat was used as a systematic analysis of cell communication based on the network analysis and pattern recognition approaches.^24^ Subsequently, we compared signalling pathways and depicted conserved and context‐specific pathways between COVID‐RS and CTRL group.

### 2.7 Viral‐Track

To systematically scan for viral RNA in scRNA‐seq data, an Unsupervised Viral‐Track Pipeline was used^25^ (https://github.com/PierreBSC/Viral‐Track).

### 2.8 HE and immunostaining

For haematoxylin and eosin (HE) staining and immunohistochemistry (IHC), tissue samples were fixed with 10% buffered formalin at room temperature for 6 h. Samples were embedded in paraffin. After routine rehydration and antigen retrieval, 5‐μm paraffin sections were stained for HE or with the primary antibodies (Table S2). The sections were further incubated with HRP‐conjugated secondary antibody and were visualized with a DAB (DakoCytomation) solution containing 0.03% H_2_O_2_. The sections were counterstained with haematoxylin and mounted. CD68 were co‐staining with Anti‐C5aR. For frozen tissue, 10‐μm transverse sections were used for immunofluorescence (IF) using Rabbit anti‐Human C3c Complement/FITC (1:10, Gene Tech) and Anti‐Cytokeratin 7 antibody (Table S2). Negative controls were performed by replacing the specific antibody with rabbit or mouse IgG. To quantify nuclear number in STB, three slides per sample were subjected to immunohistochemistry, and the number of nuclei in the STB was counted in five random fields of each slide, presented as nuclear number per square millimetre of villous area. The results were presented as mean ± SD, and statistically analysed by two‐tailed *t* test.

### 2.9 In situ hybridization

The RNAscope 2.5 Assay for in situ hybridization (ISH) from Advanced Cell Diagnostics (ACD) was performed according to the manufacturer's instructions. In brief, FFPE‐embedded tissue sections were baked on the slides at 60°C for 2 h. Each section was treated with H_2_O_2_ to quench endogenous peroxidases for 10 min at room temperature. Washed slides were boiled in RNAscope Target Retrieval Reagents for 15 min. The barriered slices were then incubated for 30 min in RNAscope Protease Plus at 40℃. The v‐nCov2019‐S probe (catalog #848561), positive probe (the Ppib probe; catalog #313911) and negative probe (targeting bacterial gene DapB; catalog #310043) were designed and synthesized by ACD. Tissues were counterstained with haematoxylin. After tapping off the excess counterstain, the slides were mounted and viewed under a microscope.

### 2.10 Western blot analysis

Whole lysates from tissues were extracted with RIPA buffer containing protease inhibitor cocktail (Sigma‐Aldrich). Protein concentrations were determined using the BCA™ Protein Assay Kit (Pierce). Western blotting was performed as described previously. Antibodies used for Western blotting include ACE2, hCG beta and β‐actin (Table S3).

### 2.11 The cytokine/chemokine profile assay

Inflammation 20‐Plex Human ProcartaPlex™ Panel (EPX200‐12185‐901, Thermo) was used to assess the concentration of cytokines in maternal and foetal plasma and placenta samples from the COVID‐RS case and CTRL group according to the manufacturer's protocol via Luminex MAGPIX system (Luminex).

## 3 RESULTS

### 3.1 Case presentation

**FIGURE 1:**
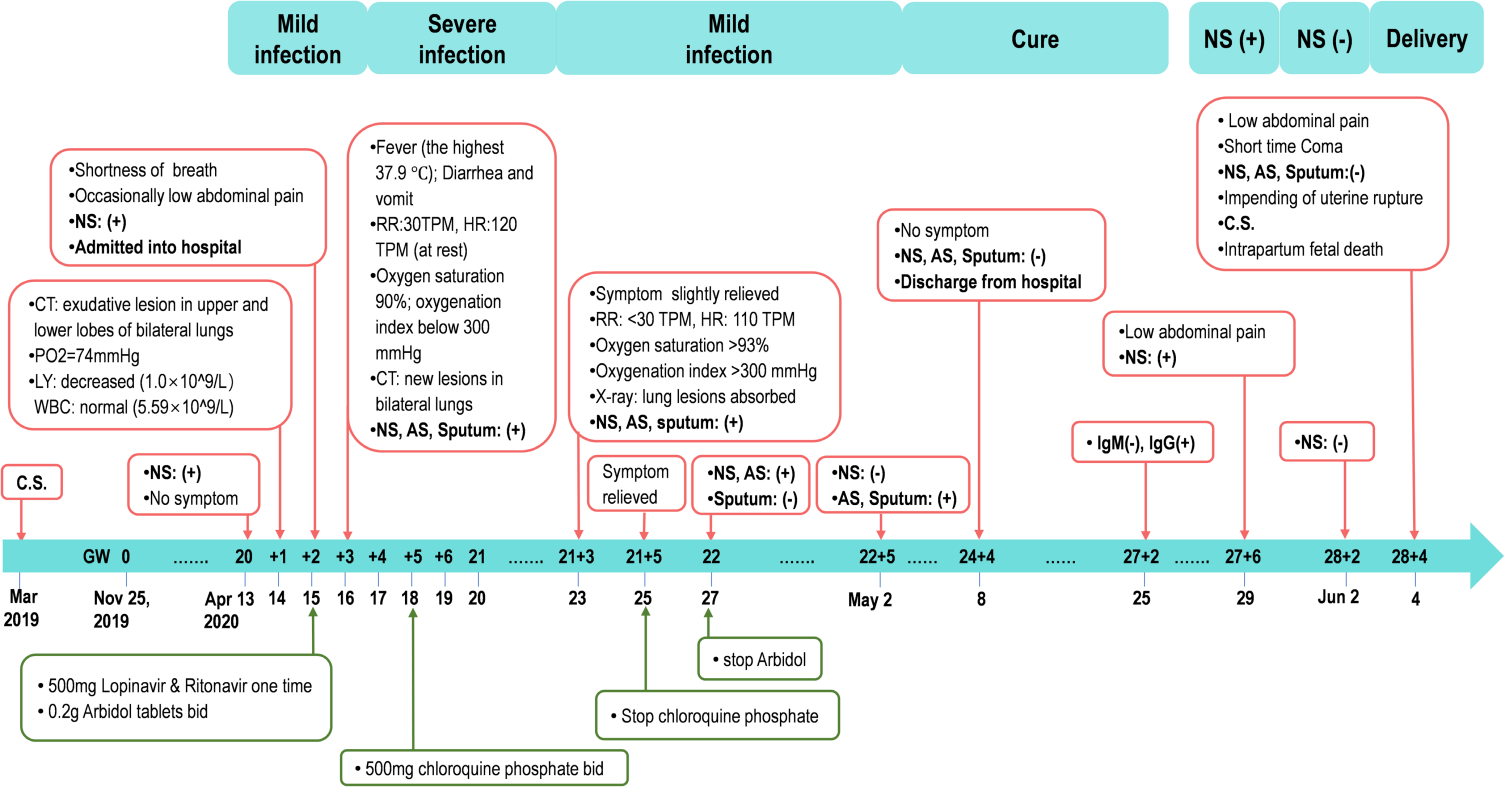
The timeline of infection and medical record in the reported case. A 30‐year‐old Chinese pregnant woman who had a previous caesarean section (C.S.) in March 2019 was diagnosed as asymptomatic SARS‐CoV‐2 infection after nasopharyngeal swab (NS) test at the 20th week of gestation (13 April 2020). Her self‐reported date of last menses was 25 November 2019. This timeline highlights the process of her SARS‐CoV‐2 infection (top) and the corresponding clinical treatments (bottom). AS, anal swab; CT, chest radiograph; HR, heart rate; LY, lymphocyte count; RR, Respiratory rate; WBC, white blood cell count

The whole process of the infection and treatment of a pregnant COVID‐19 case was illustrated in Figure 1. Due to known high‐risk exposure to a family member who had COVID‐19, a 30‐year‐old Chinese pregnant woman (gravida 3, previous parity 2), without self‐reported clinical symptoms, was detected as asymptomatic SARS‐CoV‐2 infection following her nasopharyngeal swab test at the 20th week of gestation (13 April 2020). The infection was further proved by chest radiograph, blood gas analysis and serological laboratory examination. On a subsequent day, she developed shortness of breath and occasional lower abdominal pain and was diagnosed as mild COVID‐19 according to the clinical management guidelines for COVID‐19 in pregnancy.^26^ She was admitted to the First Hospital of Foshan City, Guangdong, and was treated with antiviral therapies since April 15. Her conditions deteriorated soon and progressed to severe COVID‐19 on April 16. Combined antiviral treatment and supportive care were implemented for twelve days (from April 15 to April 27) until the clinical symptoms were remised. On May 8, she was cured and discharged from the hospital and corrected gestational age was 24^+4^ weeks by ultrasound evaluation. On May 29 (27^+6^ weeks of gestation), a routine throat swab test for SARS‐CoV‐2 on this woman showed re‐positive while without any symptoms of COVID‐19. However, her throat swab test for SARS‐CoV‐2 was negative on June 2.

On June 4 (28^+4^ weeks of gestation), she was admitted to the Emergency Department of the Third Hospital of Guangzhou Medical University due to persistent lower abdominal pain. Considering the short interpregnancy interval (IPI) of this pregnancy from her previous caesarean section (seven months) and the emergent clinical presentations, the uterine rupture was highly suspected. Termination of pregnancy was conducted following an examination and the patient's consent. Ultrasound graph showed severe foetal distress, normal placenta location and amniotic fluid volume before the caesarean section. Surgical observation showed an incomplete uterine rupture in the uterus wall around the previous caesarean incision, where muscle layer was lacking, and only uterine serosa remained and bulged outward. The foetus weighed 920 g, while died during caesarean delivery due to severe asphyxia. None apparent morphologic abnormality was observed in the foetus, placenta and umbilical cord at delivery.

### 3.2 Virological test of SARS‐CoV‐2 and histological examination of the maternal and foetal specimens

To assay SARS‐CoV‐2 infection, whole maternal blood, cord blood and amniotic fluid collected during caesarean delivery were sent for serological and virological tests. IgM to SARS‐CoV‐2 in the maternal and cord blood was negative. IgG to SARS‐CoV‐2 in maternal and cord blood tested positive, with the cut‐off index (COI) 8.04 and 4.4 respectively. The virological tests of SARS‐CoV‐2 by qRT‐PCR showed negative in all samples, including the maternal blood, cord blood, amniotic fluid, placenta and foetal tissues (brain, lung, colon, liver and thymus gland). RNAscope in situ hybridization (ISH) for SARS‐CoV‐2 RNA was carried out in the placenta and foetal tissues, and no viral RNA puncta signal was detected in these tissues (Figure S1), which was consistent with the SARS‐CoV‐2 qPCR results. In addition, mapping the reads of scRNA‐seq data of the placenta (described in detail below) to both the host reference genome and an extensive list of high‐quality viral genomes (viruSITE database, http://virusite.org/) by using Viral‐Track revealed no SARS‐CoV‐2‐related reads in the placenta cells of this case.

**FIGURE 2:**
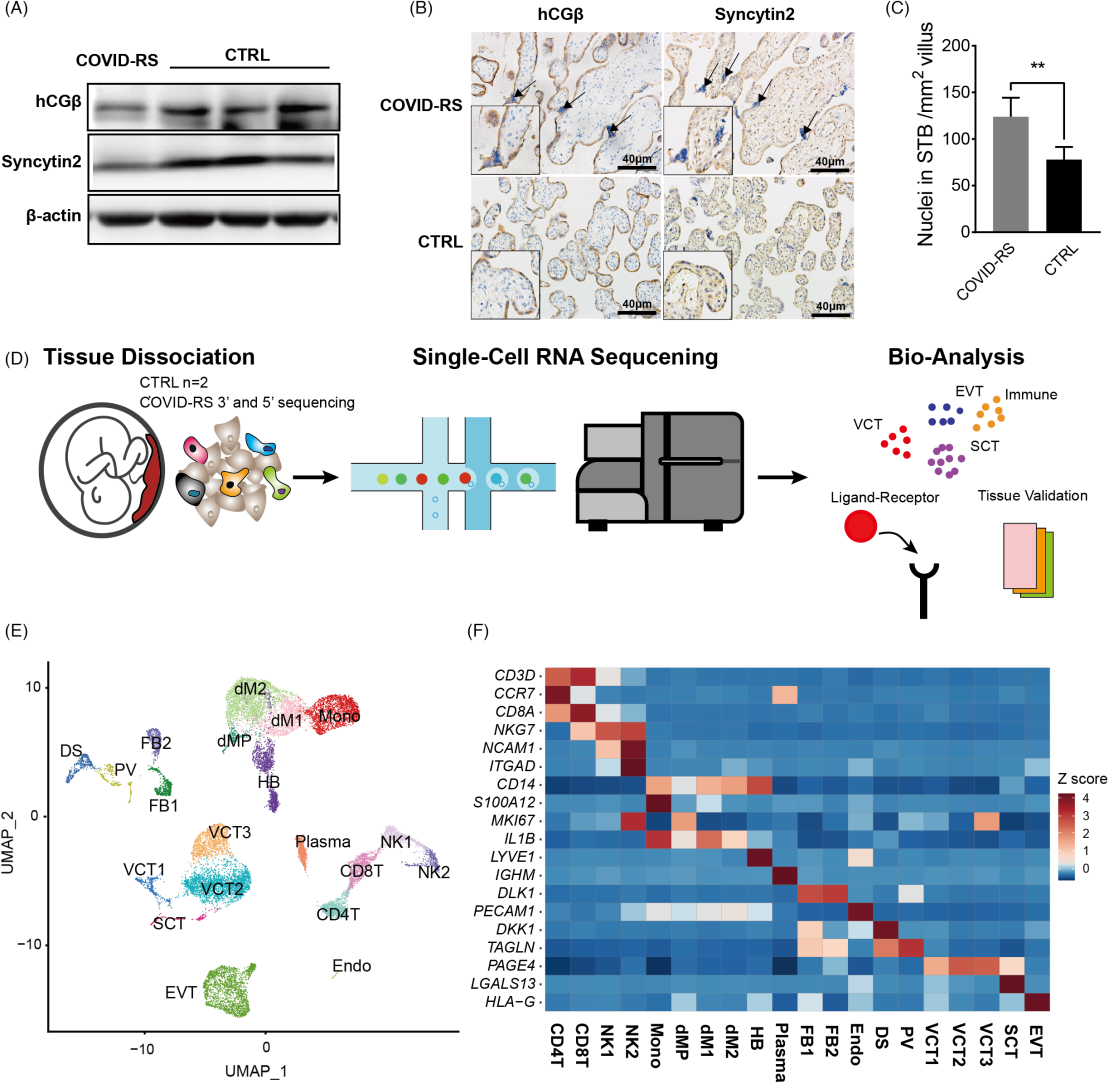
The expression of syncytialization markers and single‐cell transcription atlas in COVID‐RS placenta. (A) The expression of syncytialization markers in COVID‐RS and CTRL placenta as measured by Western blotting; (B) localization of syncytialization markers in COVID‐RS and CTRL placenta. Arrows indicate syncytial knots; (C) The number of SCT nuclei per mm^2^ villus; (D) A schematic figure illustrating the workflow of tissue collection and single‐cell RNA sequencing; (E) UMAP of the cell clustering in the placentas enrolled in this study; (F) expression of classical marker genes in each cell subsets based on the z‐score calculation

Histological examinations on the placenta and foetal tissues revealed relatively sparse villi and normal morphology of foetal organs, including the brain, lung, colon, liver and fallopian tube (Figure S2). Using preterm delivery placentas as gestational age‐matched controls (CTRL; *n* = 3, details described in Table S1), the expressions for Syncytin‐2, the marker of trophoblast syncytialization and hCGβ, the classical marker of STB secretory function, were decreased in the placenta of this case relative to the CTRL (Figure 2A,B). In addition, the COVID‐RS placenta exhibited more syncytial knots (Figure 2B,C). These observations indicated an impaired syncytialization process and inflammation response in the COVID‐RS placenta.

### 3.3 Single‐cell RNA sequencing analysis of the COVID‐RS placenta

To illustrate the molecular alterations in the maternal–foetal interface with SARS‐CoV‐2 infection at the midgestation stage, scRNA‐seq analysis was performed on the COVID‐RS placenta (5′ and 3′ sequencing, separately) and two CTRL placentas using droplet‐based 10X Genomics platform (Figure 2D). Following rigorous quality control, normalization and elimination of doublets/multiplets, a total of 17,481 high‐quality cells across these samples were classified into 20 unique cell subsets and visualized by uniform manifold approximation and projection (UMAP) (Figure 2E and Figure S3A–C). The cell subsets of trophoblasts (including villous cytotrophoblast (VCT), syncytiotrophoblast (SCT), extravillous trophoblast (EVT)), macrophages (dM1, dM2 and dMP), foetal macrophages (Hofbauer cells; HB), T cells (CD4T and CD8T), monocytes (Mono), decidual natural killer cells (dNK1 and dNK2), plasma cells (Plasma), decidual stromal cells (DS), pericytes (PV) and fibroblasts (FB1 and FB2) were identified based on the expressions of well‐defined marker genes (Figure 2F and Figure S3D).

### 3.4 Alterations in trophoblastic features indicate a placental deficiency in the COVID‐RS placenta

**FIGURE 3:**
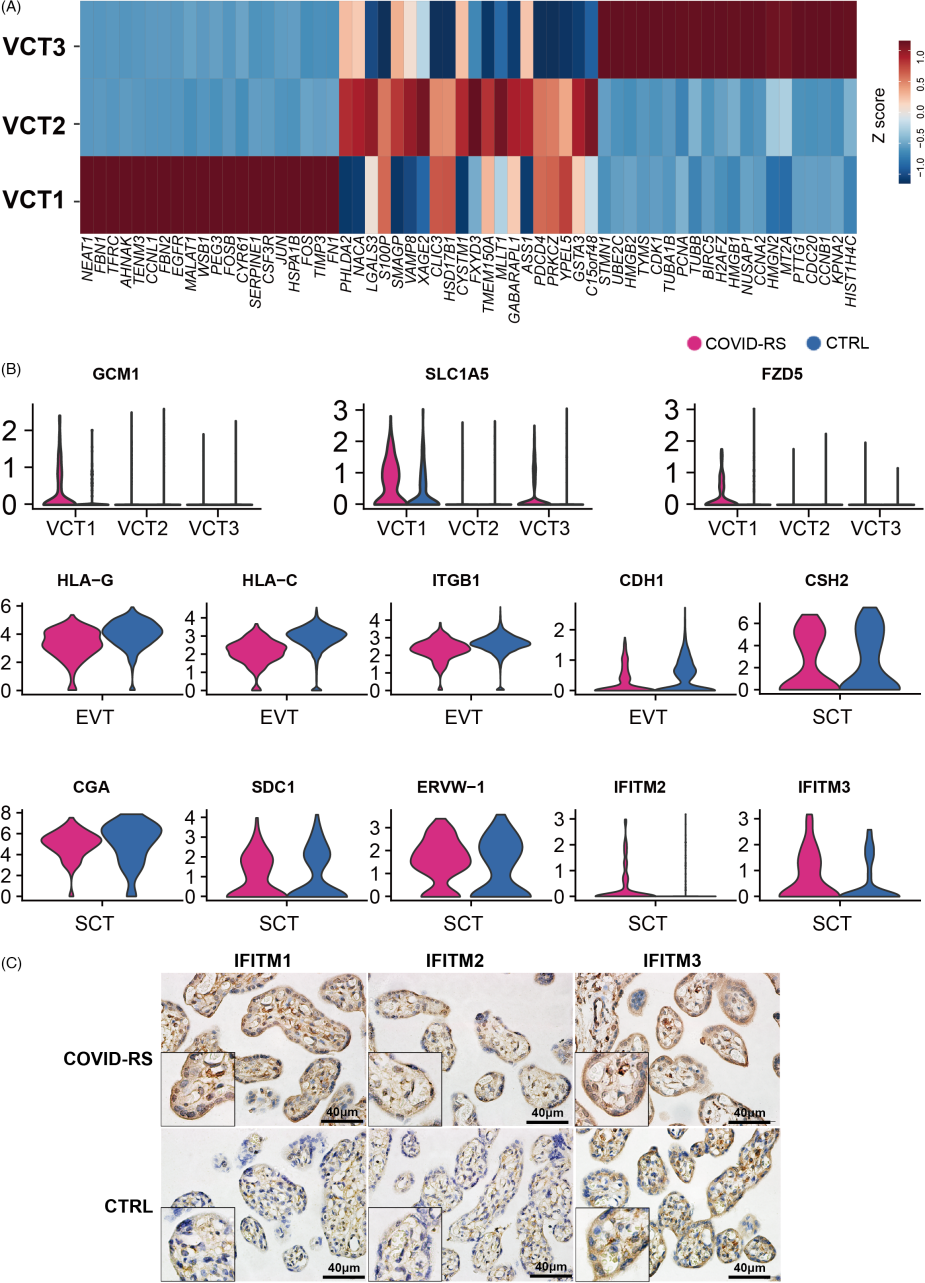
Data analysis indicating impaired trophoblast function in COVID‐RS placenta. (A) Z‐score evaluation showing the expression pattern of the top 20 genes in VCT subsets; (B) Violin plot showing the differential expression of several function‐related genes in VCT, EVT and SCT subsets between COVID‐RS and CTRL placenta; (C) immunohistochemistry for IFITM1, IFITM2 and IFITM3 in the placentas from COVID‐RS and CTRL cases

In our single‐cell sequencing data sets, 6,187 trophoblasts were identified by the expression of *KRT7* and *PERP* (Figure S3D) and were further classified into five trophoblastic subsets based on unique marker genes for each subset. The VCT cells highly expressed *GATA3* and *PAGE4*, which were clustered as three subsets. VCT1 highly expressed *SERPINE1* and *NEAT1*, inhibitors of trophoblast invasion, and specifically expressed *GCM1, SLC1A5* and *FZD5*, indicating their fate of differentiation towards the syncytial pathway (Figure 3A,B). VCT2 was annotated by higher expression of *HSD17B1* and *PHLDA2*, suggesting active functions of hormone biosynthesis (Figure 3A). VCT3 was proliferative, as shown by *PCNA* and *CDK1* genes (Figure 3A). SCT cells exhibited high expression levels of *LGALS13*, *CYP19A1* and *PSG5*, while EVT cells uniquely expressed *HLA‐G and NOTUM* (Figure 2F and Figure S3E).

Differential expression (DE) analysis showed multiple molecular changes of trophoblastic features upon SARS‐CoV‐2 infection. Compared with the CTRL group, immune tolerance–associated genes, including *HLA‐C and HLA‐G*, and cell invasion–related genes, such as *CDH1 and ITGB1*, were downregulated in the EVT subset of COVID‐RS placenta (Figure 3B). Interestingly, the expressions of *GCM1, SLC1A5* and *FZD5* were upregulated in VCT1 from the COVID‐RS placenta, indicating an active initiation of syncytial differentiation (Figure 3B). However, in the SCT subset, genes involved in syncytialization, such as *SDC1* and *ERVW‐1* (encoding Syncytin‐1), and the hormone biosynthesis genes, such as *CGA and CSH2*, were decreased in the COVID‐RS placenta (Figure 3B). In addition, IFN‐induced transmembrane proteins (IFITMs), a family of restriction factors blocking the entry and fusion step of many viruses, were recently found to inhibit syncytin‐mediated trophoblasts fusion. Here, we found markedly elevated *IFITM2* and *IFITM3* at the transcriptional level in SCT from the COVID‐RS placenta, which was supported by the immunohistochemical staining (Figure 3B,C). Although the mRNA level of ACE2 and TMPRSS2, the two functional receptors for SARS‐CoV‐2 to enter host cells, was low in the placenta, immunostaining demonstrated stronger signals for ACE2 in villous trophoblasts of COVID‐RS placenta (Figure S4A,B). Neuropilin‐1 (*NRP1*), known to significantly potentiate SARS‐CoV‐2 infectivity, displayed higher expression in dMP, dM2, HB, Endo and VCT1 subsets from COVID‐RS placenta than that from CTRL placenta (Figure S4A), which suggested strong host defence responses at the maternal–foetal interface. Together, these data indicate the dysregulated syncytial differentiation and SCT function in the COVID‐RS placenta may be partially caused by upregulation of IFITMs, which is induced by robustly activated interferons pathways and lead to compromised trophoblast fusion.

### 3.5 Antiviral and pro‐inflammation response in COVID‐RS placenta

**FIGURE 4:**
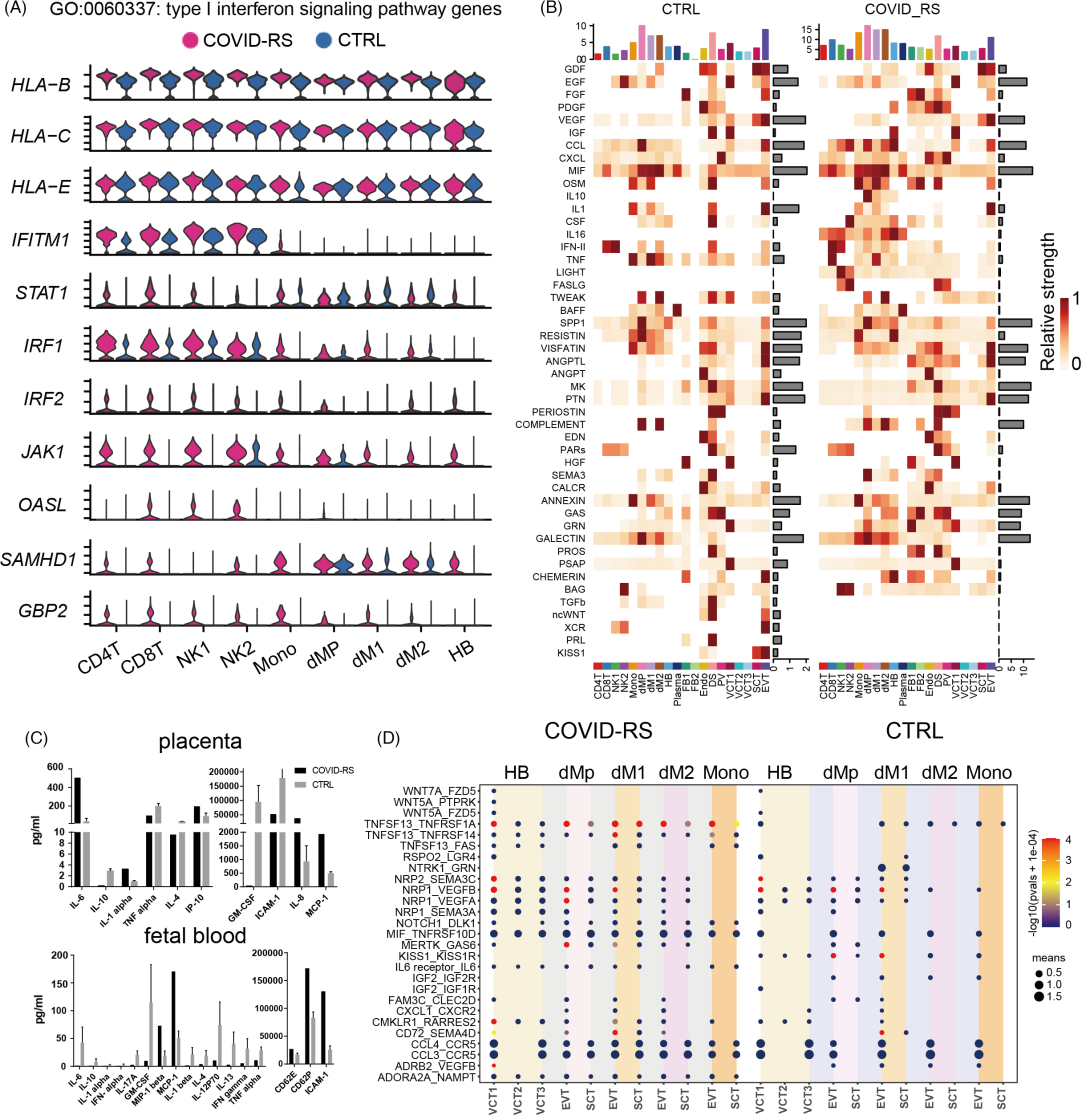
Analysis of sc‐RNAseq data indicates the inflammation response in the COVID‐RS placenta. (A) Violin plot showing the expression of IFN signalling‐related genes in immune cell subsets from COVID‐RS and CTRL placenta; (B) the signalling pathway analyses by Cellchat in all cell subsets from COVID‐RS and CTRL placenta; (C) the cytokine/chemokine profile in the placenta tissues and umbilical cord blood samples from COVID‐RS and CTRL cases; (D) CellphoneDB analysis showing the ligand–receptor interactions among macrophage, monocytes and trophoblast subsets in COVID‐RS and CTRL cases. The size of dots represents mean value and the colour shows −log(pvals +1e‐04)

We found the antiviral response in immune cell subsets. The upregulated genes in dNK1, dM1, dM2, CD4T and CD8T subsets were involved in viral gene expression and viral process‐related pathways in the COVID‐RS placenta (Figure S5A). CD8T lymphocytes displayed the characteristic transcriptional profile of antiviral response with high expression of TNF and PRF1 (encoding perforin) (Figure S5B,C). We also found type I interferon (IFN) signalling pathway was enriched among all immune subsets in the COVID‐RS placenta. Given the importance of the IFN response to induce the expression of various interferon‐stimulated genes (ISGs) that confer antiviral activities to host cells, we evaluated the expression of interferon signalling pathway‐related genes in each immune cell type between COVID‐RS and CTRL (Figure 4A). *IFITM1, IRF1, IRF2, JAK1, OASL, SAMHD1* and *GBP2* were upregulated in CD4T, CD8T, NK1, NK2 and monocyte subsets from COVID‐RS, and *STAT1, IRF2, JAK1, SAMHD1* and *GBP2* were upregulated in macrophage subsets including foetal‐derived HB cells from COVID‐RS (Figure 4A), indicating that the activation of antivirus response in maternal‐ and foetal‐derived immune cells.

Appropriate interaction between trophoblasts and other cell types at the maternal–foetal interface establishes the microenvironment supporting foetal growth. Here, the overall signalling patterns in the placenta by Cellchat showed extensive enrichment of inflammation‐related signalling pathways, such as IL‐16, FASLG, IL‐10, CCL and CXCL in COVID‐RS placenta (Figure 4B and Figure S7A). Dot plot of pro‐inflammatory cytokines revealed higher transcriptional levels of inflammatory‐related genes, such as *CXCL8, CCL2, IL‐1B, IL‐10, IFITM1, TLR4, TNFRSF1B* and *IRF1*, in macrophages subsets (Figure S6A) of the COVID‐RS placenta (Figure S6B).

A further cytokine/chemokine profile assay in the placenta tissues using Inflammation 20‐Plex Human ProcartaPlex™ Panel demonstrated a significant increase in the production of IL‐6, IL‐1α, IP‐10 (encoded by *CXCL‐10*), IL‐8 and MCP‐1 (encoded by *CCL2*) in the COVID‐RS placenta (Figure 4C and Figure S6C). Interestingly, results of the multiplex assay also showed higher productions of MCP‐1 (encoded by *CCL2*), MIP‐1 beta (encoded by *CCL4*), CD62E, CD62P and ICAM‐1 in cord blood from the COVID‐RS case compared to the corresponding CTRL (Figure 4C and Figure S6C), which indicates profoundly changed inflammatory responses in the fetoplacental unit.

Moreover, as revealed by CellphoneDB analysis, there existed significant alterations in the potential crosstalk between trophoblasts and DS, FB and macrophage in COVID‐RS placenta compared to CTRL (Figure S7B). Specifically, inflammation‐related cytokine–receptor interactions, such as CCL3‐CCR5, CCL4‐CCR5, CMKLR1‐RARRES2, IL6R‐IL6, TNFSF13‐TNFRSF1A and TNFSF13‐TNFRSF14 between trophoblasts and macrophage, were significantly promoted in COVID‐RS placenta (Figure 4D). Meanwhile, ligand–receptor pairs contributing to trophoblast growth and differentiation, such as KISS1‐KISS1R, PRL‐PRLR, WNT5A‐FZD1/3/5 between DS/FB and trophoblasts, IGF2‐IGF1R/IGF2R, KISS1‐KISS1R, NTRK1‐GRN between macrophages and trophoblasts, were significantly declined in the COVID‐RS placenta (Figure 4D and Figure S7C). In parallel, noncanonical WNT (ncWNT), PRL and kisspeptin/KISS1R pathways involved in trophoblast development were attenuated in the COVID‐RS placenta by Cellchat analysis, and the expression of critical ligand–receptor pair molecules among those pathways decreased in the placenta of this case (Figure S7A,D).

Taken together, these data indicate the potentially impaired interactions between trophoblasts and other cells at the maternal–foetal interface and the elevated pro‐inflammatory response in the COVID‐RS placenta, which potentially causes pro‐inflammatory status in the foetus.

### 3.6 Over‐activation of complement in the COVID‐RS placenta

**FIGURE 5:**
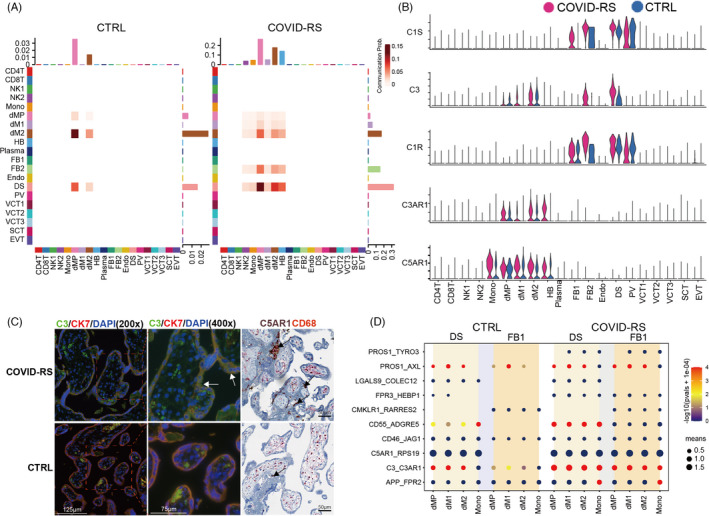
Data analysis showing complement over‐activation in COVID‐RS placenta. (A) Cellchat analysis showing complement signalling networks among different cell subsets in COVID‐RS and CTRL cases. The Receiver and Sender are listed at X and Y axis respectively; (B) Violin plot showing the expression of several critical ligand–receptor pairs in the complement pathway, including C1R, C1S, C3, C3AR1 and C5AR1, in all subsets from COVID‐RS and CTRL placenta; (C) Immunofluorescence or immunohistochemistry showing the activated C3 deposition in the COVID‐RS placenta. Villous trophoblasts and macrophages are labelled by the staining for CK7 and CD68 respectively. The black arrows shows the accumulation of C5aR1‐expressing macrophages; (D) CellphoneDB analysis showing the complement activation–related ligand–receptor pairs among subsets in COVID‐RS and CTRL placenta. The size of dots represents mean value, and the colour shows −log(pvals +1e‐04)

Complement has been well known to promote immune cell activation and the pro‐inflammatory states, and complement over‐activation is associated with severe pregnancy complications. Recent emerging evidence suggests the critical role of complement activation in the SARS‐CoV‐associated acute respiratory distress syndrome (ARDS).^27^ Applying Cellchat analysis on our scRNA‐seq data, we found an over‐activation of the complement pathway in the COVID‐RS placenta (Figure S7A), with DS and FB cells as the primary driving sources of complement ligands to target macrophage subsets (Figure 5A). As shown by the violin plot illustrating the expression of critical ligand–receptor pair genes in the complement pathway, complement‐related genes including C1R, C1S and C3 were mainly expressed in DS and FB cells, whereas complement receptor genes such as C3AR1 and C5AR1 were enriched in macrophages (dM1, dM2 and HB) and monocytes (Figure 5B). Immunostaining results validated significant accumulation of the activated C3 deposition in villi and increased C5AR1 intensity in CD68‐labelled macrophages in the COVID‐RS placenta (Figure 5C). Our CellphoneDB analysis showed that complement activation‐related ligand/receptor pairs between DS/FB1 and macrophages were significantly increased in the COVID‐RS placenta (Figure 5D).

## 4 DISCUSSION

Here, we reported a whole COVID‐19 disease process in a pregnant woman infected with SARS‐CoV‐2 at the second trimester, covering stages from asymptomatic, mild illness, severe illness, recovery, to pregnant termination due to an incomplete uterine rupture. Combined with the scRNA‐seq of the placenta, we provided for the first‐time systematic cellular and molecular evidence indicating placental insufficiency and potential impacts on the maternal and foetal health that may be attributed to antiviral and pro‐inflammatory responses induced by SARS‐CoV‐2 infection at the midgestation stage in a recovered COVID‐19 pregnant woman.

Recent studies reported that around 13.55%–88% of pregnant women exhibit asymptomatic infection for SARS‐CoV‐2,^28^, ^29^ and 10% of asymptomatic pregnant women have developed to the symptomatic stage. In our study, the woman was admitted into the hospital at the asymptomatic stage, which ensures a well monitor of her disease process and timely treatment, including antiviral medicine and oxygen support, the commonly used therapeutics for COVID‐19 pregnancy. It has been reported that pregnant women with severe or critical COVID‐19 display a high rate of acute respiratory distress syndrome (ARDS),^30^ while the patient in our study did not show signs of ARDS after she developed severe symptoms, most likely because of the timely therapies which greatly shortened the duration of the severe ill stage. In addition, the symptoms of this case were soon relieved following effective anti‐infection treatment without causing significant foetal tissue abnormalities. Therefore, the regular test of SARS‐CoV‐2 for pregnant women with high exposure risk is recommended, and closely monitoring, timely diagnosis and therapeutic interventions can effectively decrease the adverse impacts of SARS‐CoV‐2 on the mother and the foetus.^26^

Although our study and others’ reports demonstrated the expressions of SARS‐CoV‐2 entry receptors,^31^, ^32^ such as ACE2 and NRP1 in human placenta, only a few cases showed transplacental transmission of SARS‐CoV‐2, perhaps due to the high viral load and the timing of maternal infection.^8^, ^11^, ^12^ In our reported case, SARS‐CoV‐2 infection at midgestation did not cause vertical transmission, although the patient underwent the severe stage of COVID‐19. It is well known that the placenta plays a critical role in defending against intrauterine infection and preventing vertical transmission. Thus, it is most likely that a relatively well‐developed placental structure could efficiently reduce the risk of vertical transmission of SARS‐CoV‐2 in the second trimester. In addition, rare intrauterine infection with SARS‐CoV‐2 owed to the fine‐immune defence/modulation mechanisms, such as the interferon (IFN) family and potent physical barrier in maternal–foetal interface.^33^ Lu‐Culligan reported that cellular subsets at the maternal–foetal interface demonstrate significantly increased expression of interferon‐related genes in individuals with COVID‐19 compared with healthy control individuals.^16^ Similar to this study, interferon signalling pathway‐related genes were upregulated in the COVID‐RS placenta in our data, especially in NK and T subsets.

Meanwhile, we also found decidua CD8^+^ T cells, which have a mixed profile of T cells and retain the capacity to respond to pro‐inflammatory events,^34^ were activated by high expression of TNF, PRF1 and interferon signalling pathway‐related genes in the COVID‐RS placenta. The action of the antiviral machinery in the placenta could protect the foetus from in utero infection.^35^, ^36^ The antiviral interferons of type I (IFN‐α) and type III (IFN‐λ) inhibit SARS‐CoV‐2 replication.^37^, ^38^ Inborn errors of TLR3‐ and IRF7‐dependent type I IFN immunity underlie life‐threatening COVID‐19 pneumonia, also suggesting the antiviral effect of the type I IFN for SARS‐CoV‐2.^39^ Thus, timely antivirus treatment and the antivirus response in the placenta may help to prevent vertical transmission of SARS‐CoV‐2 to the growing foetus.

In this case, we were unable to follow through with the maternal and foetal outcomes until late gestation because the pregnancy was terminated due to uterine rupture caused by a short IPI from the last caesarean section. We also did not find apparent histopathological abnormalities in the foetus from this COVID‐RS case, except for a relatively low foetal weight^40^ indicating potential foetal growth restriction. However, our systematic analysis of the placenta strongly indicates potential problems in maternal and foetal health. First, we found the syncytialization markers, such as SDC1, Syncytin‐1 and Syncytin‐2, and the hormone biosynthesis function, such as hCGβ, were decreased in STB, while the syncytial trophoblastic knots representing an apoptotic end‐stage of the STB life cycle^41^ were increased in the COVID‐RS placenta. Abnormal formation or function of STB during pregnancy may hinder material and gas exchange between the mother and the foetus and thus impair foetal growth, implicating in the aetiology of pregnancy complications, such as preeclampsia and foetal growth restriction.^42^ This may be the reason for the low foetal weight in this COVID‐RS case. In addition, *HLA‐C* and *HLA‐G* in the EVT subset, which is critical to establish protective immunity at the maternal–foetal interface, are downregulated in COVID‐RS placenta, indicating the impairment in immune tolerance to the foetus. Several key pathways that regulate EVT invasion, such as WNT, PRL and kisspeptin pathways,^43^ are depressed in this COVID‐RS placenta, which may lead to insufficient remodelling of uterine spiral arteries and thus insufficient blood perfusion. As indeed, several studies reported the occurrence of preeclampsia‐like symptoms in pregnant patients who had severe forms of COVID‐19.^7^, ^44^ IFITM proteins, induced robustly by IFNs, which are identified as viral restriction factors that inhibit infection mediated by Marburg virus (MARV), EBOV and SARS‐CoV,^45^ inhibit placental syncytiotrophoblast formation.^46^ The high expression of IFITMs in trophoblasts from the COVID‐RS placenta would explain, at least in part, the placenta damage after maternal SARS‐CoV‐2 infection.

Second, the notable pro‐inflammatory state in the COVID‐RS placenta indicates potential harm to foetal outcomes. Macrophages are one of the main leukocyte groups present throughout pregnancy and are a source of homeostasis as well as inflammatory responses to infection.^47^ In our case, macrophages in the COVID‐RS placenta expressed higher transcript levels of inflammatory‐related genes, such as *CXCL8* and *IL*‐*1B*, indicating that an inflammatory status was still present in the placenta although SARS‐CoV‐2 was absent. The placental inflammatory response, specifically IL‐1β, induced by viral infections, is known to increase the risk of perinatal developmental abnormalities.^48^ Further, compared with the transcriptional response induced by a high viral load of SARS‐CoV‐2 in placenta,^49^ the similar expression pattern of innate antiviral immunity and strong chemotactic and inflammatory responses factors in the COVID‐RS placenta showed that the immune responses in the placenta to viral infection might persist for long periods although the case was recovery.

Third, the features of the activated complement system may contribute to triggering the inflammatory response in the COVID‐RS placenta. It has been found that most COVID‐19 patients with respiratory failure present consistent over‐activation of complement system^50^ and high expression of C5AR1 receptors in pulmonary myeloid cells.^51^ Generally, complement inhibition at the fetomaternal interface is essential for pregnancy success. Here, in the COVID‐RS placenta, several complement‐associated genes were upregulated in DS and FBs, in parallel with extensive C3 deposition in the placenta and higher expression of C5AR1 receptor in macrophages. The dysregulated complement activation has been indicated in the pathogenesis of pregnancy complications such as preeclampsia and preterm birth.^52^ Consistent with the pro‐inflammatory status in the placenta, we also found elevation of inflammatory factors, such as MIP‐1 beta (CCL4), MCP‐1(CCL‐2), CD62E, CD62P and ICAM‐1, in the foetal blood of this case. ICAM‐1, CD62E and CD62P are reported to be regulated by complement activation and drive complement attack, in association with various diseases.^53^, ^54^ Consistently, a previous report also found an increase in pro‐inflammatory molecules in newborns from SARS‐CoV‐2‐infected mothers.^11^ These findings indicate that the intrauterine inflammatory response caused by SARS‐CoV‐2 infection during pregnancy may lead to foetal inflammation and potential long‐term adverse outcomes, such as neurodevelopmental disorders.^55^ Although the foetus does not display apparent pathological change in many tissues in our reported case, it is of importance to study whether offspring are at high risk of health problems if the pregnancy continues.

By far the evidence on the long‐term outcomes of pregnant women who had suffered from COVID‐19 is relatively scarce. In a study of 675 pregnant women admitted for delivery, postpartum readmission differed among women with symptomatic COVID‐19 (6.7%), asymptomatic infection of COVID‐19 (3.6%) and without COVID‐19 infection (1.5%).^56^ In comparison, Ko et al. reported no significant influence by COVID‐19 status on readmission rate within 30 days of delivery.^57^ Despite this, the severity of COVID‐19 greatly impacts gene expressions in immune cells even at the recovery phase, as revealed by the single‐cell sequencing of blood samples taken at 56 and 119 days after the symptom onset, irrespective of pregnancy status.^58^ Our findings are in consistence with this report, showing higher inflammatory responses in maternal cells from the COVID‐RS case, indicating a unique feature in both innate and adaptive immune responses in the COVID‐RS placenta.

Additionally, we recognize that the main limitation of our study is the lack of an appropriate control group. In this study, gestationally age‐matched nonexposed placentas delivered due to cervical incompetence were chosen as the control group. Although the ideal control is the gestationally age‐matched, nonexposed, and with uterine rupture placenta, clinically it is extremely difficult to obtain such controls. Our present findings are consistent with the recent report from Lu‐Culligan's study of the placental transcriptome in COVID‐19 cases, showing significant activation of SARS‐CoV‐2 infection–related immune genes, including complement factors and interferon‐stimulated genes. These studies together indicate local inflammatory response to systemic SARS‐CoV‐2 infection in the placenta of SARS‐CoV‐2‐infected pregnant women.

So far, there has been limited evidence regarding the follow‐up of maternal and foetal health in women suffering from COVID‐19, especially being infected at early‐to‐mid gestation. Nonetheless, our findings in the placenta of this case demonstrate defects in trophoblasts and their interactions with other uterine cells, as well as antiviral response, pro‐inflammation response and complement over‐activation at the fetomaternal interface from the recovered COVID‐19 pregnancy. These cellular and molecular changes have been highlighted to affect foetal development and the short‐term or long‐term health of the offspring. Early screening of asymptomatic infection, timely clinical treatment and extensive monitoring for pregnant women should be essential strategies to reduce the harm to the infected mothers and newborns. In addition, a long‐term follow‐up study in a larger population will warrant an in‐depth understanding of the long‐term consequences of COVID‐19 pregnancy and their offspring.

## CONFLICT OF INTEREST

There are no competing financial or nonfinancial interests regarding this work.

## AUTHOR CONTRIBUTIONS

D.J.C, Y.L.W. and B.C. jointly conceived and supervised the project, interpreted the data and revised the manuscript. J.S.C followed up the patient, interpreted the clinical data and participated in draft preparation. L.L.D. and F.Y.W performed scRNA‐seq and data analysis and drafted the manuscript. X.S. and W.Z.Y performed bench analysis of the placenta and participated in data analysis. X.Y.W, D.X.C, X.F. P, R.F.C, F.W.X, X.Y. L, S.P.W, M.L.Z, M.X.L., L.L., H.Y., F.H. and L.Y. managed the patient and collected clinical information. S.S.Z, L.J.H. and Y.L.L helped in sample collection and processing. S.L.B, L.Z.Z and Y.Y.L participated in data analysis. Q.P.J and Z.T.X. performed the pathological examination and clinical data interpretation. All authors have read and approved the final manuscript and declared no conflict interests.

## Supporting information

Figure S1

Figure S2

Figure S3

Figure S4

Figure S5

Figure S6

Figure S7

Table S1–S3

## Data Availability

The data sets generated and analysed in the study are available in the Genome Sequence Archive (HRA000830) and can be accessed upon request. All custom scripts can be accessed upon request to the corresponding author.
