# Supplementary figures and images for: Cellular and molecular atlas of the placenta from a COVID‐19 pregnant woman infected at midgestation highlights the defective impacts on foetal health
Source: Cell Prolif. 2022 Feb 9;55(4):e13204. doi: 10.1111/cpr.13204 (PMC9055894; doi:10.1111/cpr.13204)

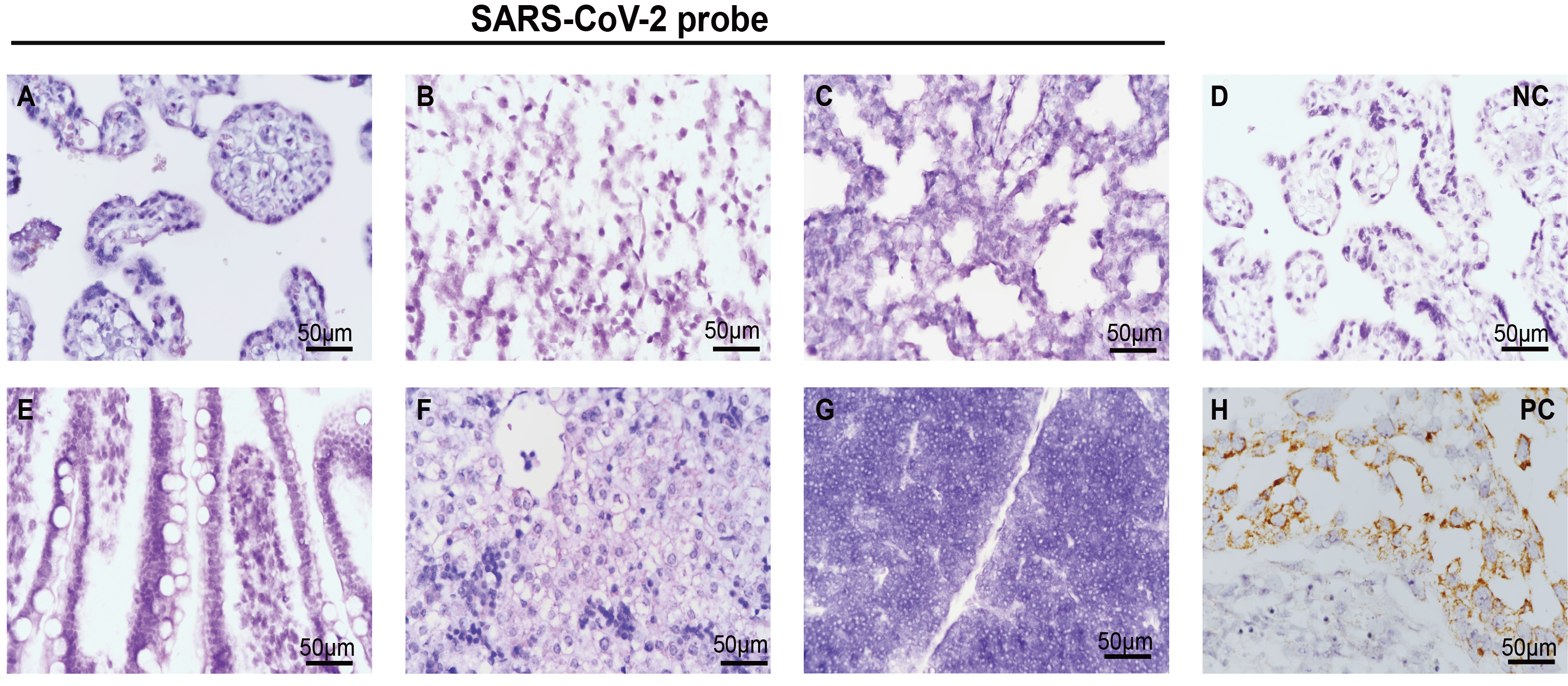

Supplement: Supplementary file 1 — Figure S1 [file CPR-55-0-s004.png]

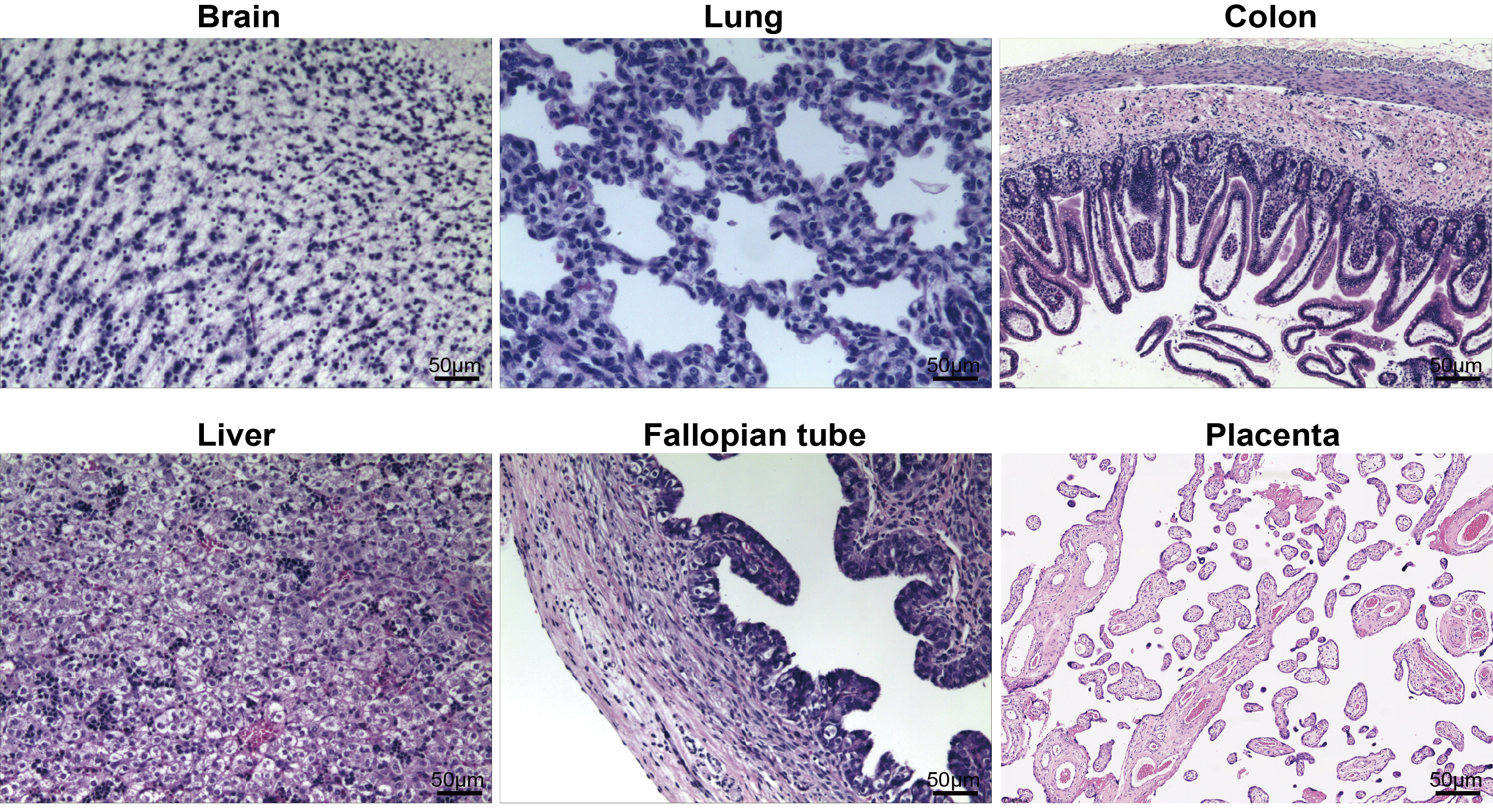

Supplement: Supplementary file 2 — Figure S2 [file CPR-55-0-s006.png]

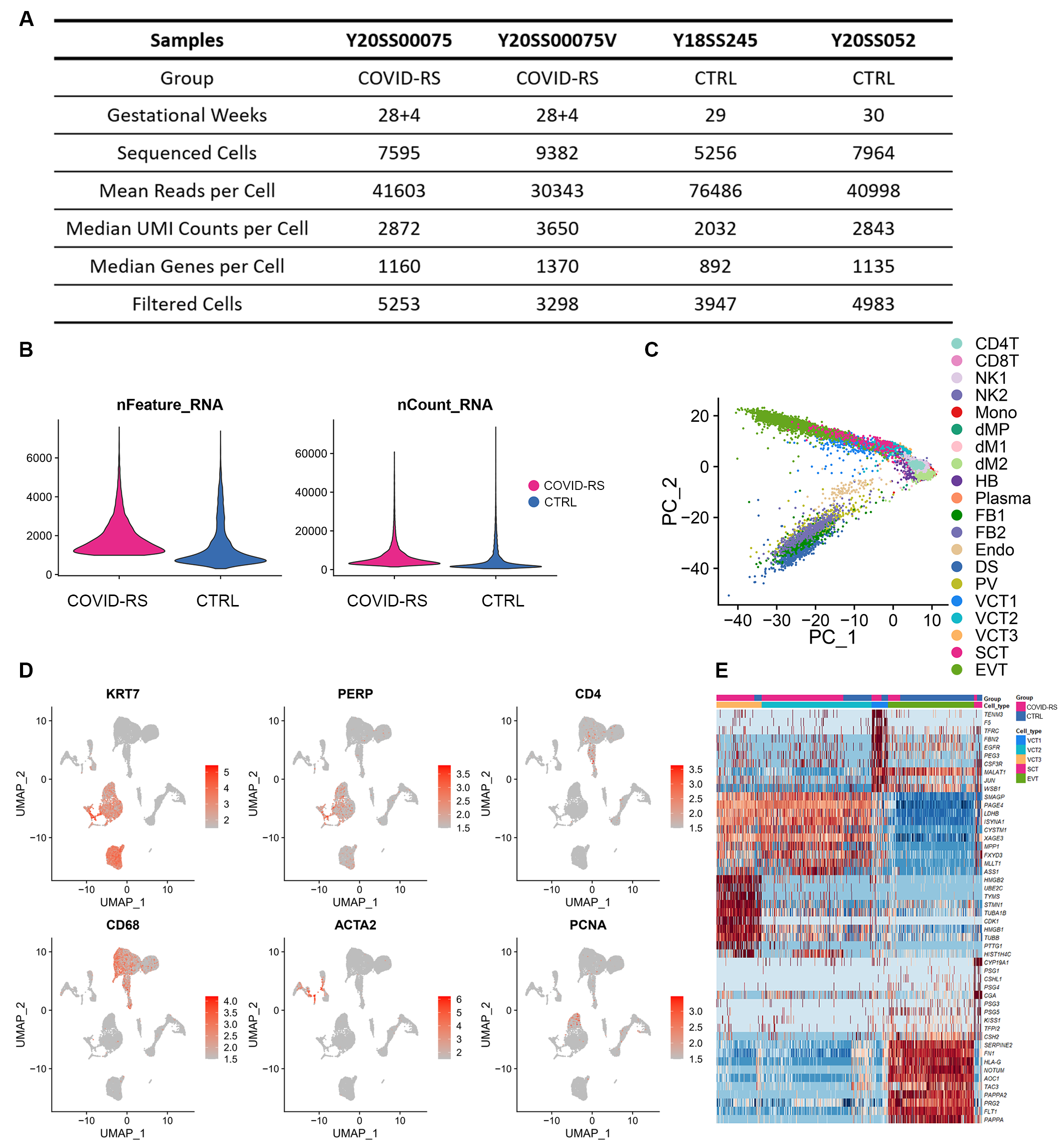

Supplement: Supplementary file 3 — Figure S3 [file CPR-55-0-s003.png]

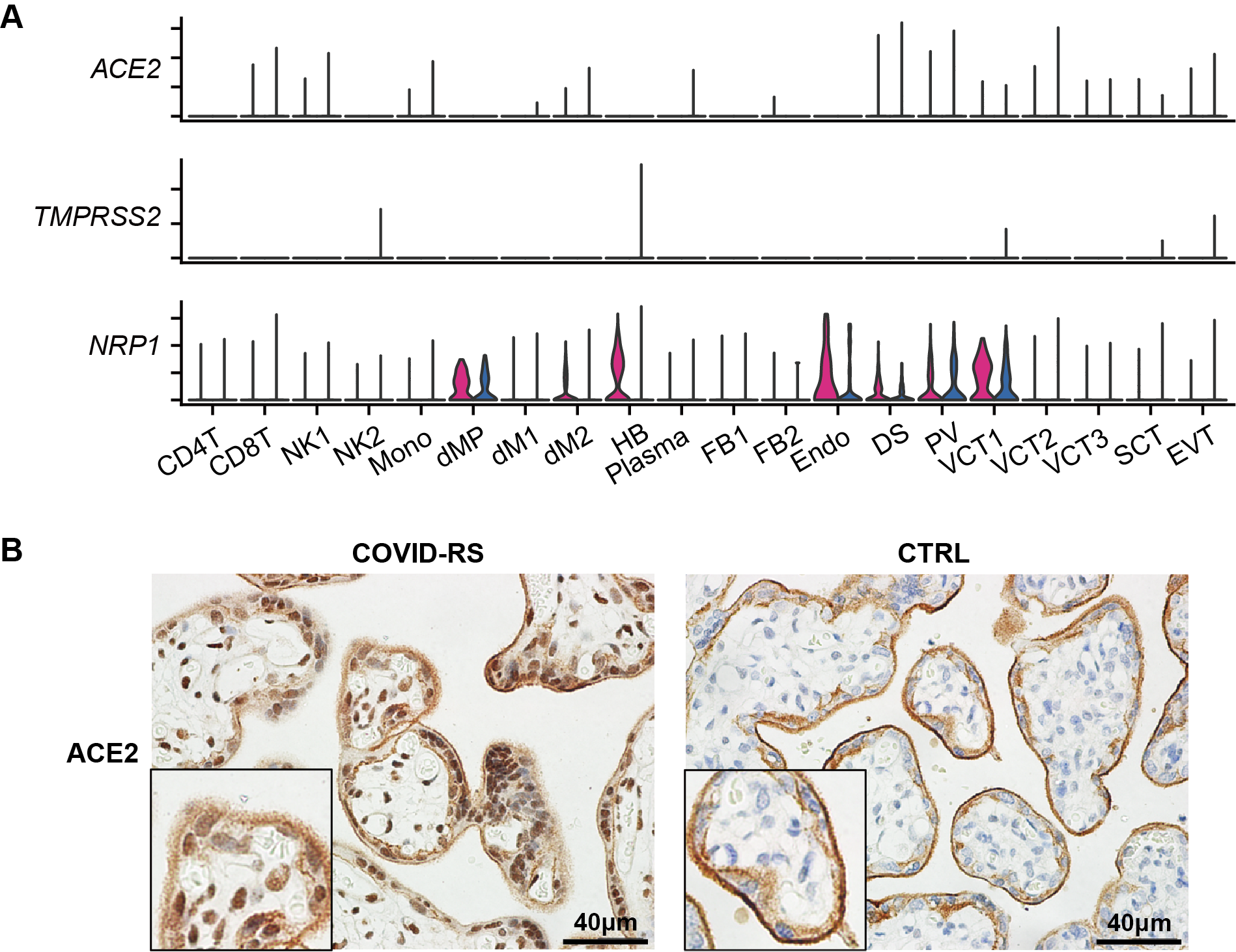

Supplement: Supplementary file 4 — Figure S4 [file CPR-55-0-s001.png]

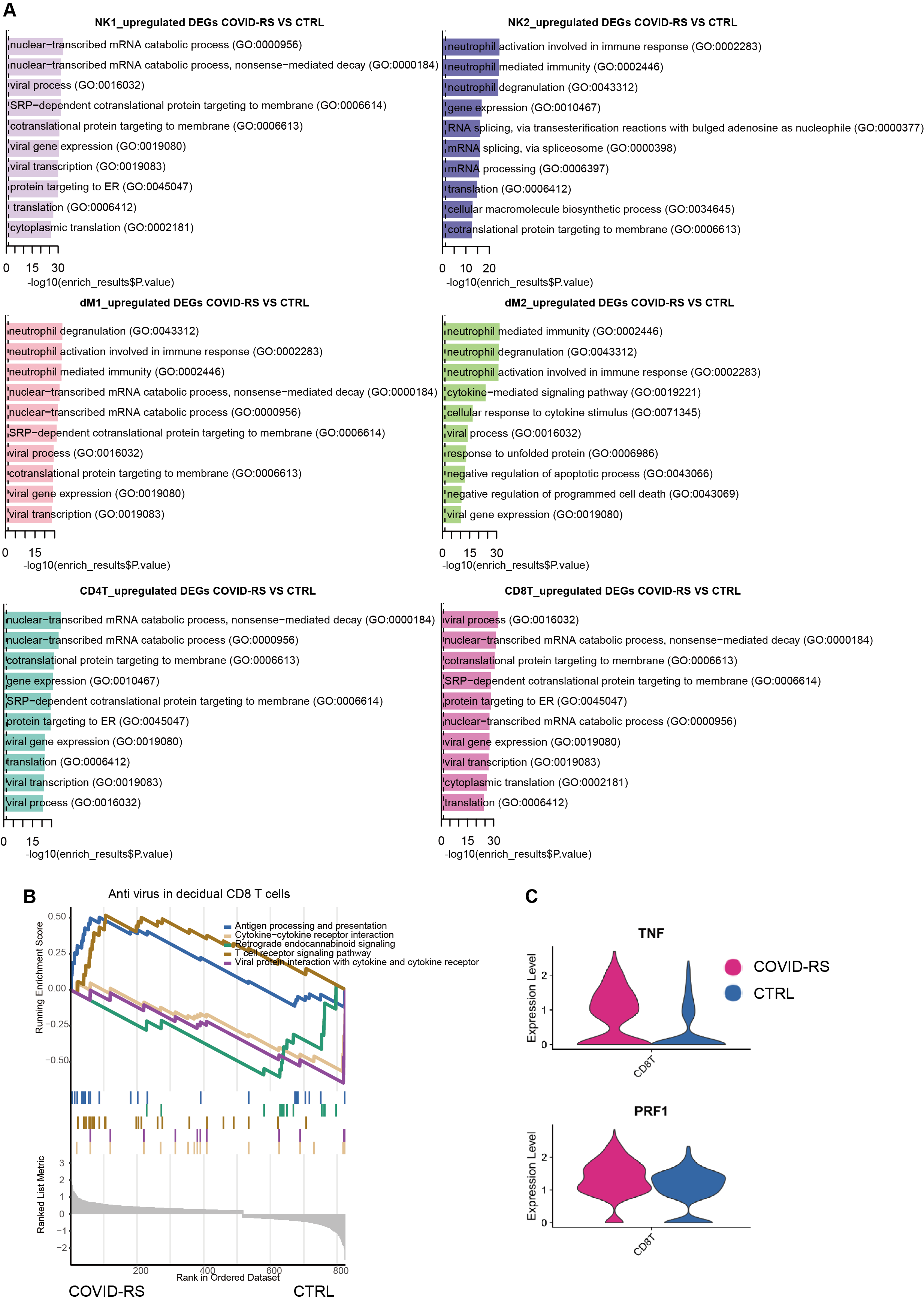

Supplement: Supplementary file 5 — Figure S5 [file CPR-55-0-s005.png]

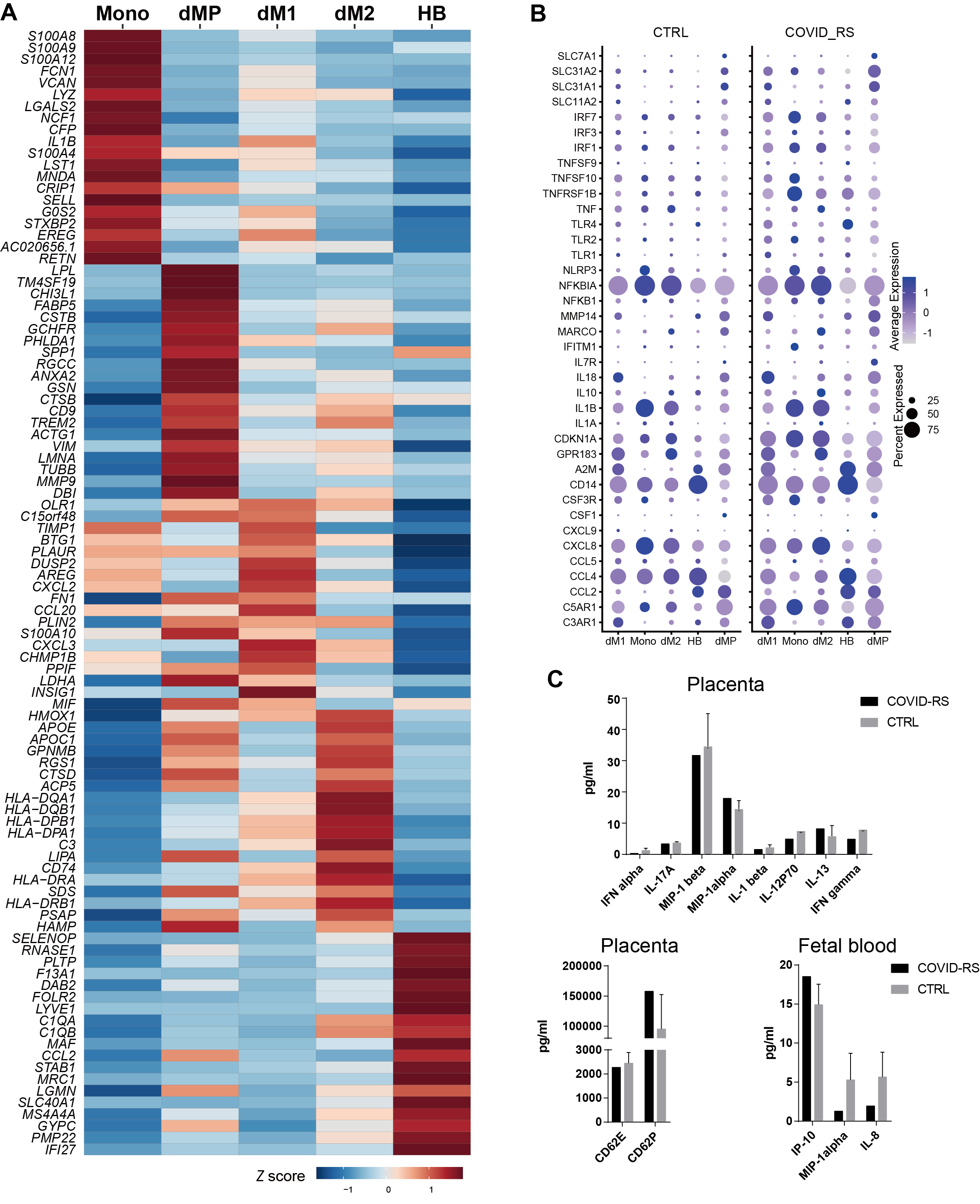

Supplement: Supplementary file 6 — Figure S6 [file CPR-55-0-s002.png]

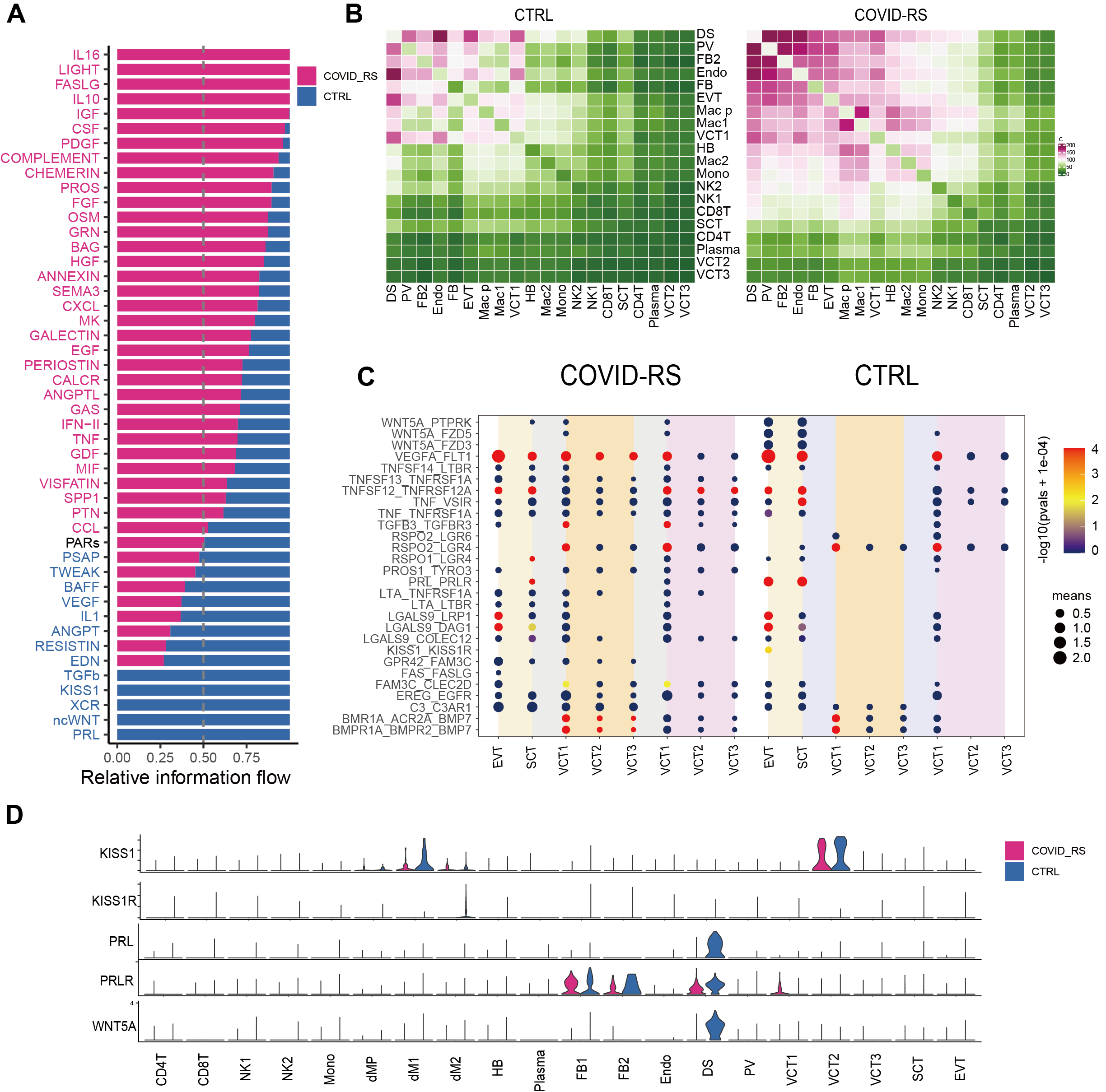

Supplement: Supplementary file 7 — Figure S7 [file CPR-55-0-s008.png]
