# Supplementary material for: Cellular and molecular atlas of the placenta from a COVID‐19 pregnant woman infected at midgestation highlights the defective impacts on foetal health
Source: Cell Prolif. 2022 Feb 9;55(4):e13204. doi: 10.1111/cpr.13204 (PMC9055894; doi:10.1111/cpr.13204)
Supplement: Supplementary file 8 — Table S1–S3 [file CPR-55-0-s007.docx]

**Supplemental Table 1 Clinical characteristics of the pregnant women enrolled in this study.**


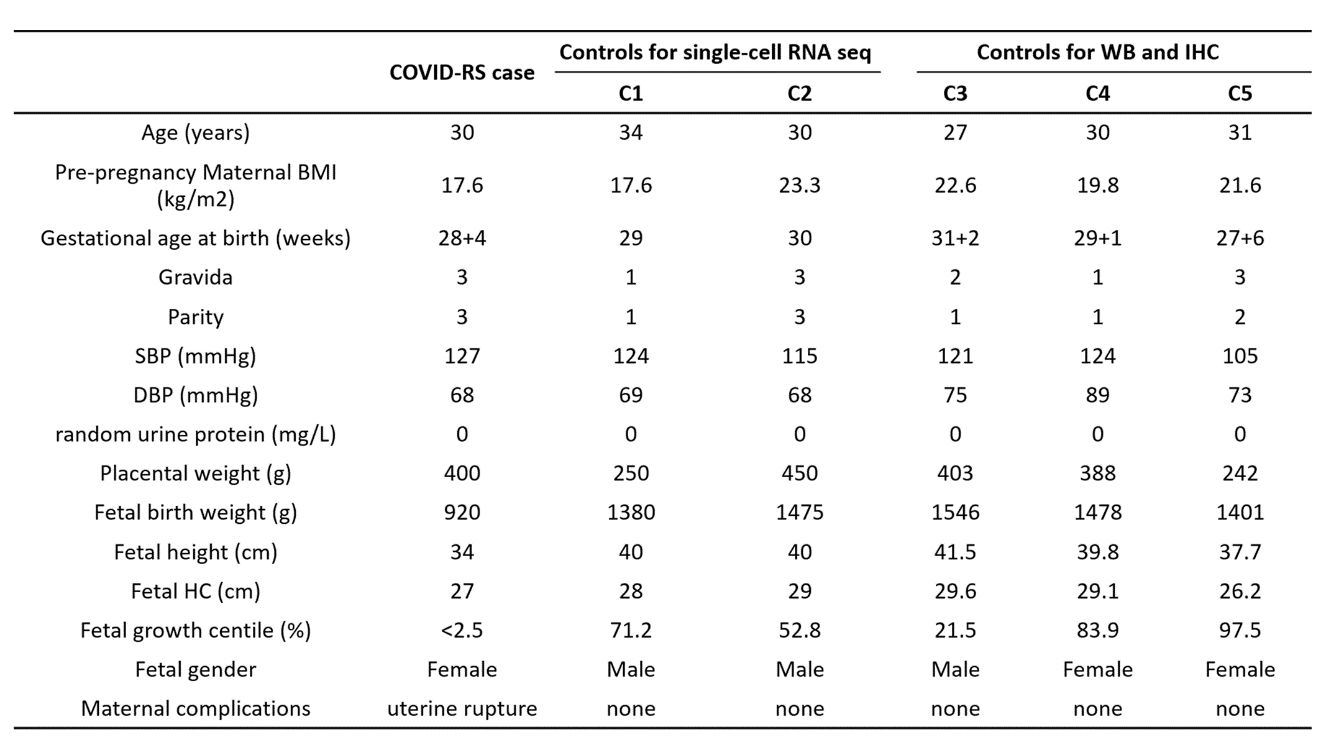
Note: The number of Gravida and Parity included this pregnancy.

Fetal growth centile was calculated according to the data in NICHD Fetal Growth Studies for Asian singleton fetuses (Buck Louis GM, Grewal J, Albert PS, Sciscione A, Wing DA, Grobman WA, Newman RB, Wapner R, D’Alton ME, Skupski D, Nageotte MP, Ranzini AC, Owen J, Chien EK, Craigo S, Hediger ML, Kim S, Zhang C, Grantz KL. Racial/ethnic standards for fetal growth: the NICHD Fetal Growth Studies. Am J Obstet Gynecol. 2015 Oct;213(4):449.e1-449.e41).

**Supplementary Table 2** Antibodies used in IHC and IF of this study.

| Antibody | Host species | Final concentration | Manufacturer | Catalogue number |
| --- | --- | --- | --- | --- |
| ACE2 | Rabbit | 1:200 | Abcam | ab108252 |
| Syncytin2  Anti-C5a-R | Rabbit  Rabbit | 1:200  1:500 | Abcam  Abcam | ab171382  ab252435 |
| hCGβ | Mouse | 1:200 | ZSGB-BIO | ZM-0134 |
| β-actin | Mouse | 1:200 | CST | #3700 |
| C3-FITC | Rabbit | 1:10 | Gene Tech | GF020129 |
| CK7 | Rabbit | 1:100 | Abcam | ab181598 |
| Secondary Antibody,  Alexa Fluor 594 | Donkey  anti-Rabbit | 1:500 | Invitrogen | A-21207 |

CST: Cell Signaling Technology

**Supplementary Table 3** Antibodies used in Western blotting of this study.

| Antibody | Host species | Final concentration | Manufacturer | Catalogue number |
| --- | --- | --- | --- | --- |
| ACE2 | Rabbit | 1:1000 | Abcam | ab108252 |
| Syncytin2 | Rabbit | 1:1000 | Abcam | ab171382 |
| hCGβ | Mouse | 1:1000 | Abcam | ab9582 |
| β-actin | Mouse | 1:1000 | CST | #3700 |

CST: Cell Signaling Technology
